# Rehabilomics: A state-of-the-art review of framework, application, and future considerations

**DOI:** 10.3389/fneur.2023.1103349

**Published:** 2023-03-08

**Authors:** Wenyue Cao, Xiuwei Zhang, Huaide Qiu

**Affiliations:** ^1^Faculty of Rehabilitation Science, Nanjing Normal University of Special Education, Nanjing, China; ^2^Rehabilitation Medicine Center, The First Affiliated Hospital of Nanjing Medical University, Nanjing, China

**Keywords:** rehabilomics, biomarkers, personalized treatment, functional evaluation, rehabilitation mechanisms

## Abstract

Rehabilomics is an important research framework that allows omics research built upon rehabilitation practice, especially in function evaluation, outcome prediction, and individualized rehabilitation. In the field of rehabilomics, biomarkers can serve as objectively measured indicators for body functioning, so as to complement the International Classification of Functioning, Disability, and Health (ICF) assessment. Studies on traumatic brain injury (TBI), stroke, and Parkinson's disease have shown that biomarkers (such as serum markers, MRI, and digital signals derived from sensors) are correlated with diagnosis, disease severity, and prognosis. Rehabilomics also examines a wide range of individual biological characteristics in order to develop personalized rehabilitation programs. Secondary prevention and rehabilitation of stroke have already adopted a rehabilomic approach to individualize treatment programs. Mechanisms of non-pharmacological therapies are expected to be unveiled in light of rehabilomics research. When formulating the research plan, learning from established databases is recommended and a multidisciplinary collaborative team is warranted. Although still in its infancy, the advancement and incorporation of rehabilomics has the potential to make a significant impact on public health.

## Introduction

Rehabilitation offers services for populations who differ in individual impairments, functional limitations, and response to treatments. Rehabilitation practitioners tailor programs to clients based on their specific needs, individual dysfunctions, and personal factors, thereby making standardized protocols not optimal for maximizing individual outcomes. The issue of individual variability comes to the fore when verifying the therapeutic effect of a particular intervention or clinical decision (1). Differences in rehabilitation outcomes may be attributed to different rehabilitation regimens, biological effects of therapies, and patient responsiveness. Therefore, researchers proposed that an individual's biological characteristics (biomarkers) can be used to tailor a personalized approach to rehabilitation, termed “rehabilomics” (2, 3). Essentially, this biomarker-based concept provides an omics integration for the study of rehabilitation processes and outcomes, providing personalized rehabilitation programs designed to optimize individual outcomes (4).

Biomarkers are a group of objectively measured indicators of physiology, pathogenic processes, or response to treatments (5). In most areas of omics research, biomarkers identified in blood and other body fluids are typically considered. However, in the context of rehabilomics research, medical imaging and sensor measurements can also be candidate biomarkers. The identification of biomarkers generally includes differential analysis, correlation analysis, predictive model, and prognostic model (Table 1). The present article aims to review the framework, applications, and challenges of rehabilomics, thereby providing a reference for researchers in this field.

**Table 1:** General process of identifying biomarkers.

Differential analysis	Identification of markers by differences between cases and controls
Correlation analysis	The correlation between the level of the marker and certain functions
Predictive model	Prediction of the patient's response to a certain therapy
Prognostic model	Prediction of the patient's functional outcome

## Rehabilomics and International Classification of Functioning, Disability and Health (ICF)

The World Health Organization (WHO) proposed ICF in 2001 (6), which incorporated three domains (body function and structures, activity, and participation) in human functioning. ICF is a classification designed to organize and record information about functioning and disability (7). However, ICF has more than 1,400 categories, and its applicability warrants optimization. Although some studies have proposed simplified forms of ICF such as generic set (8), core set (9), and rehabilitation set (10), the objectivity, accuracy, and repeatability of ICF assessment are still major challenges. With the development of omics and big data technology, we may be able to use biomarkers to measure the functional level of individuals in the future. Biomarkers have the advantages of objectivity, high measurement accuracy, and good repeatability, which can serve as a powerful supplement for functional evaluation. Linking individual dysfunction to biomarkers is central to the concept of rehabilomics, and combining biomarkers with rigorous design and data collection is essential to rehabilomics research. Wagner (4) proposed that incorporating the ICF model into the identification of rehabilitation-related biomarkers will contribute to the report of patient-centered results and promotion of function-oriented practice. The rehabilomics model was adapted from the ICF on the relationship between injury, activity, and participation. The model describes how the biology underlying individual characteristics evolving from environmental exposures affects the physiological environment, leading to disease and its complications, and ultimately to impaired functioning and compromised quality of life. The rehabilomics model also considers how these individualized physiological factors interact with other individual factors in the ICF model to affect functioning.

## Biomarkers for functional evaluation

Numerous studies have shown that serum biomarkers can predict the risk of cognitive and behavioral dysfunctions in traumatic brain injury (TBI). Serum hormones, inflammatory markers, and neurotrophic factor levels can predict fatigue (11), depression (12, 13), behavioral problems (14), and cognitive deficits (12, 15) after TBI, which are associated with impaired self-care capacity and quality of life. There is a strong association between the levels of cell surface markers characterizing neuroinflammation [soluble intracellular adhesion molecule (sICAM), soluble vascular adhesion molecule (sVCAM), and soluble Fas (sFAS)] in the acute phase and depression 6 months after moderate to severe TBI (12, 13). Specifically, individuals with high levels (>75%) of these three biomarkers had a positive predictive value of 85.7% for post-traumatic depression at 6 months. In addition, both acute and chronic TNF-α (Tumor necrosis factor-α) levels were associated with suicidal tendencies at 6 months (14). Serum levels of Brain-Derived Neurotrophic Factor (BDNF) within the first week after brain injury were associated with functional memory scores at 6 and 12 months after injury; lower serum BDNF levels in the early post-injury period were associated with poor memory scores (12, 15). Likewise, elevated serum cortisol levels, measured within the first week of moderate-to-severe TBI, were also associated with poor functional outcomes and performance on neuropsychological cognitive tests (cognitive composite score and functional Independence Measure–Cognition) 6 and 12 months after injury (12, 15). The above studies show that these biomarkers predict the prognosis of TBI patients and can serve as a supplement to the existing evaluation indicators.

Notably, the biomarkers in rehabilomics are not limited to microscopic molecules such as gene expression, proteins, and metabolites. Emerging studies using medical imaging, electrophysiological indicators, and sensor data in rehabilomics have become a new research focus. Omics research based on medical imaging data is called “Radiomics” (16). Magnetic resonance imaging (MRI) data, which can reflect the structure of the brain and the activation of specific brain regions, are commonly-used markers in neurorehabilitation research. It can be used to measure neuroplasticity in patients with cerebral palsy (17), monitor the efficacy of stroke rehabilitation (18), and predict the treatment response to transcranial magnetic stimulation (TMS) in stroke survivors (19). Studies have shown that MRI texture analysis contributes to the early diagnosis of ischemic stroke (20), and imaging quantitative analysis of the penumbra can predict the short-term prognosis of acute ischemic stroke (21), while MRI white matter hyperintensity is associated with long-term mortality from ischemic stroke (22). MRI data can also be used to study mechanisms of recovery in Parkinson's disease (23) and traumatic brain injury (24), which is likely to be applied in other dysfunctions.

With the popularization of wearable devices, digital biomarkers collected by sensors have gradually gained attention. Built-in sensors such as accelerometers and gyroscopes in mobile devices are capable of converting motion characteristics into digital signals, which can serve as quantitative surrogate indicators of diagnosis and assessment of Parkinson's disease (25, 26). Digital biomarkers, which can be collected remotely and transmitted in real-time with little interference in daily life, have broad application scenarios in the mobile internet era. Using machine learning or deep learning techniques such as support vector machines (27, 28) and convolutional neural networks (29), diagnostic models for PD were established on these smartphone-based gait data, finger touches, and typing time series data. Meanwhile, sensor-based data can not only classify different motor symptoms such as bradykinesia, tremor, and dyskinesia in Parkinson's disease but can also be used to assess the severity of these symptoms (30). Similar application can be found in other conditions. For instance, bioelectrical signals collected from wearable devices such as electroencephalogram (EEG) and Transcranial Doppler are enabling the soild objective data to help the clinicians evaluate functional impairments of mTBI (mild traumatic brain injuries) (31). In another study, three novel digital biomarkers including convergence points (CP), physical activity (PA), and functional range of motion (fROM) were devised to evaluate the longitudinal, bilateral movement, and allow for personalized therapy schedule in hemiparetic patients. This study indicated the advantage of digital biomarkers in the recovery process (32). The data collected from accelerometers revealed the positive association between more sedentary behaviors and worse physical function in a large epidemiological study including 1,168 knee osteoarthritis patients (33).

## Personalized rehabilitation based on biomarkers

Rehabilomics is a biomarker-centered framework that not only complements existing evaluation metrics but also examines a wide range of individual biological characteristics to develop individualized rehabilitation programs. Taking stroke as an example, rehabilomics research has provided new insights for stroke prevention and rehabilitation. In the secondary prevention of stroke, the commonly used antiplatelet drug clopidogrel needs to be converted into its active metabolite by hepatic cytochrome p450 (CYP) to take effect, and decreased effects of clopidogrel were observed in carriers of CYP2C19 loss-of-function allele (34). However, up to 25% of White patients and 60% of Asian patients carry this genotype, which leaves clopidogrel a bad choice for the subgroups. In comparison, ticagrelor does not require metabolic activation for its antiplatelet effect, and therefore similar or greater levels of inhibition of platelet aggregation can be expected (35). The results of the multi-center clinical trial CHANCE-2 (ClinicalTrials.gov identifier: NCT04078737) showed that in Chinese patients with mild ischemic stroke or TIA carrying the CYP2C19 loss-of-function allele, the ticagrelor (+aspirin) has a modestly lower 90-day stroke risk than clopidogrel (+aspirin) with no significant increase in the risk of moderate to severe intracranial bleeding (36). The use of ticagrelor plus aspirin regimen for secondary stroke prevention reflects the value of omics research to guide individualized and precise therapy.

**Figure 1:**
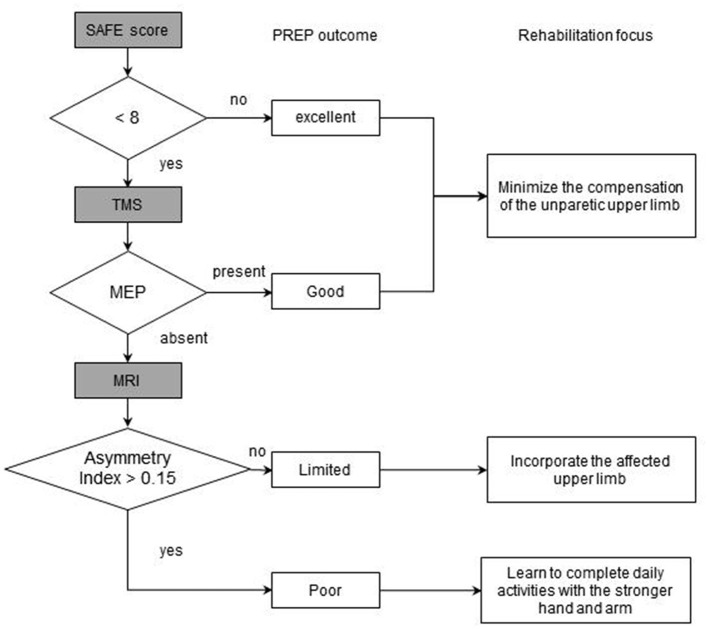
The flowchart of PREP model.

Stinear et al. (37) proposed the PREP model (Predicting REcovery Potential) for upper-limb rehabilitation in stroke, which combines muscle strength on shoulder abduction and finger extension (SAFE), TMS motor-evoked potentials, and MRI asymmetry metrics. As shown in Figure 1, in the cases with SAFE < 8, TMS was utilized to ascertain whether the MEPs (motor evoked potentials) of paretic upper limb can be elicited. The asymmetry index was calculated by MRI to make the eventual classification if the MEPs were absent. Thus, the function of the upper limb on the affected side 6 months after stroke onset was predicted, and the patients were divided into four categories according to their recovery potential, and the corresponding intervention focus was formulated (37). In patients with good or excellent outcome, minimizing the compensation of the unparetic upper limb is the focus; whereas, incorporating the affected upper limb should be the main strategy in patients with limited predicted outcome. In the meantime, learning to complete daily activities with the stronger hand and arm is encouraged in cases with poor outcomes (Figure 1). It was reported that the implementation of the PREP algorithm leads to reduced length of stay without compromising rehabilitation outcomes (37).

Due to the modifiability of the gut microbiome, its application in rehabilitation settings has also gained research interests (38). Multiple clinical studies have found 62 up-regulated (e.g., Streptococcus, Lactobacillus, Escherichia) and 29 down-regulated microbial taxa (eg, Eubacterium, Rosella) in stroke patients compared with healthy controls (39). Consumption of foods rich in choline and L-carnitine such as red meat (40) produces trimethylamine N-oxide (TMAO) is positively associated with stroke (41). However, intervention studies targeting microbiota for stroke have mainly focused on animal models and no reports are presented in clinical settings. Clinical intervention targeting gut microbiota and its derived metabolites may provide new strategies for stroke prevention and treatment.

## Biomarkers for revealing mechanisms of rehabilitation therapy

With advances in the biomedical field, rehabilomics research may contribute to the identification of non-pharmacological therapeutic mechanisms. Immune cells in the tumor microenvironment have become the basis for a new paradigm of exercise therapy for cancer. Researchers have found that voluntary movement induces an influx of immune cells into tumors and reduces tumor incidence and proliferation by more than 60% in several mouse models (42). The most responsive immune cells to exercise are natural killler (NK) cells, which were observed in the circulation in large numbers during physical activity (43). The mobilization of NK cells mediated by exercise is very rapid; NK cells increase 6-fold 70s after climbing stairs (44), and the maximum mobilization of NK cells can be achieved after 30 min of endurance training, which lasts for 3 h (45). During exercise, muscle-derived actin, increases in body temperature, and intratumoral vascularization and perfusion induce regulation, redistribution, and activation of NK cells. These activities of NK cells correlate with reduced tumor growth (46, 47). It is worth noting that more and more clinical trials of cancer rehabilitation use biomarkers such as serum inflammatory markers, hormones, and cytokines as secondary outcome indicators of clinical trials to reveal relevant mechanisms (Table 2).

**Table 2:** Clinical trials for revealing relevant mechanisms in rehabilitation.

**Clinical trials**	**ClinicalTrials.gov ID**
The effects of exercise on cardiovascular health in patients with prostate cancer	NCT03776045
Effects of exercise on metastatic breast cancer	NCT04120298
Effects of an exercise intervention on physical activity during chemotherapy in early breast cancer patients	NCT02159157

## Future considerations in advancement of rehabilomics

In the authors' opinions, the appropriate timing for using biomarkers in clinical decision-making depends on what the specific decision is, namely diagnosis, prognosis, or treatment strategies. When examining the diagnosis, the use of biomarkers certainly is as early as possible to acquire the information needed. Likewise, the investigation of predicting function outcomes (prognosis) requires early use of biomarkers (if any), as further time since the onset of a condition introduces increasingly more factors that contribute to function. These early stages often involve rapid changes in the underlying disease processes, biomarkers may provide critical information for early diagnosis (20–22) and prognosis (11–15, 37). However, in the later stages of a condition, biomarkers could be as useful in monitoring disease activity (25, 26, 30) and treatment response (19, 48) for adjusting treatment accordingly. Simpkins holds the opinion that omics can be pursued during each phase of diseases (49), which is consistent with ours. The appropriate timing for using biomarkers will ultimately depend on the specific biomarker and the condition under examination. Data from wearables could be more applicable in chronic phases of various conditions, where treatment/changing the biology itself is not the goal and the goal shifts to improving function. Biofeedback intervention based on these digital biomarkers are currently investigation, which is also known as digital medicine (50–53).

Several limitations in the application of biomarkers in rehabilitation need to be considered. One limitation is the lack of specificity, as some biomarkers can be elevated or altered due to other conditions or factors, leading to false positive or false negative results. For example, heartrate variability has been associated with a number of conditions/symptoms (54), and may be too unspecific or variable across people to be useful clinically (55). A significant challenge is how to choose the most relevant one among the massive biomarkers. Notably, a single biomarker may not be sufficient, where panels of biomarkers could serve as better indicators (56). Another limitation is variability, as the measurement of biomarkers can be affected by factors such as age, sex, diet, physical activity, and medications, leading to differences in results between individuals. Additionally, the cost of measuring biomarkers can limit their accessibility, especially for low-income families. Furthermore, the lack of standardization in methods used to measure different biomarkers can lead to difficulties in comparing results across studies or patient populations (57). Finally, the same biomarkers could yield contradictory results (58, 59) due to heterogeneity of clinical profiles, sample size, and different methods for measurements. Therefore, extensive validation in clinical studies are needed before the putative biomarkers can be used to inform clinical decision-making in rehabilitation.

When formulating a rehabilomics research plan in clincal studies, multiple issues must be considered, including (1) Construction of a research framework including a real-world cohort with the rigorous design of acquisition variables (including omics data, functional variables, etc.); (2) Measurement and collection of biomarkers including genetics, epigenetics, microbiomics, metabolomics, medical imaging, and sensor data; (3) Infrastructure required for large-scale longitudinal studies and long-term follow-up information, involving the establishment and maintenance of databases; (4) Standardization of analysis workflow for informatics, including imaging information based on expertise and data characteristics. To ensure the reproducibility of rehabilomics research, three basic elements including data management, analysis process, and algorithm code need to be considered (60). To our knowledge, data management is one of the most important. To better manage data, we refer to relatively mature databases, such as The Cancer Genome Atlas (TCGA) (61), The Gene Expression Omnibus (GEO) (62), and Medical Information Mart for Intensive Care IV (MIMIC-IV) database (63). TCGA is a landmark cancer genomics initiative that has so far molecularly characterized more than 20,000 primary cancers and matched normal samples covering 33 cancer types. GEO includes gene expression profile data including tumor and non-tumor diseases with more than 800,000 samples incorporated. The MIMIC-IV is a critical care database of more than 40,000 patients admitted to the ICU of Beth Israel Deaconess Medical Center. Relevant health-related data, including demographics, physical measurements, laboratory tests, procedures, medications, caregiver records, imaging reports, and mortality (including post-discharge). The structure and analysis process of these established databases set examples as to how to deal with local data for rehabilomics research. In the upcoming 5G era, the application scenarios of rehabilomics may be further enhanced through the development of telerehabilitation and point-of-care detection platforms to support clinical data collection for use in conjunction with rehabilitation-related biomarkers.

The identification of rehabilitation-related biomarkers often requires the use of machine learning algorithms to establish models by screening features. Some commonly used machine learning algorithms in rehabilitation such as support vector, Random Forest Logistic Regression (LR) has been of value in rehabilitation evaluation. Toyohiro Hamaguchi et al. developed a support vector machine (SVM)-based classifier to train and test the kinematics-related parameters of peak angle and peak velocity in stroke patients with finger movements impairment. High separation accuracy was obtained when predicting the classification of paralytic movements (64). Domenico Scrutinio et al. predict the 3-year mortality of patients with severe stroke through the implementation of synthetic minority oversampling technique and the Random Forests. The finding suggested that the machine learning algorithms have the higher accuracy compared with traditional regression models in predicting outcome (65).

In the framework of machine learning, there are generally two datasets, one is used to train the model, and the other is used to test or verify the performance of the model (66). Based on this research paradigm, only markers that have been extensively validated in external validation sets have stable and reliable diagnostic, predictive, or prognostic value. In this regard, real-world studies (RWS) conducted in real medical settings, especially with large samples, are preferred. Compared with randomized controlled trials (RCTs), the eligibility criteria for RWS patients are relatively loose, and the conclusions have more generalizability, thereby avoiding the realistic trap of difficulty in implementation. In the authors' opinions, a multidisciplinary collaborative team that includes clinical and translational researchers, biostatisticians, and rehabilitation experts is needed for the translation of research findings.

## Conclusion

Rehabilomics is a theranostic approach that exploits biomarkers for evaluation of rehabilitation process/outcomes, as well as for personalized treatment programs and identification of therapeutic mechanisms. Although still in its infancy, the advancement and incorporation of rehabilomics has the potential to make a significant impact on public health.

## Author contributions

WC and XZ contributed to the manuscript writing and revision. HQ conceived the study, conducted literature review, and wrote the initial draft. All authors contributed to the article and approved the submitted version.
